# Osteonecrosis as a rare musculoskeletal complication in Behcet’s disease- the largest case series with literature review

**DOI:** 10.1186/s41927-023-00366-3

**Published:** 2023-11-30

**Authors:** Mohammad Nejadhosseinian, Mazyar Babagoli, Seyedeh Tahererh Faezi, Hoda Haerian, Farhad Shahram, Majid Alikhani, Fereydoun Davatchi

**Affiliations:** 1 Joint Reconstruction Research Center, Tehran University of Medical Sciences, Tehran, Iran; 2 Rheumatology Research Center (RRC), Tehran University of Medical Sciences (TUMS), Tehran, Iran

**Keywords:** Behcet disease, Osteonecrosis, Corticosteroid therapy, Vasculitis

## Abstract

**Background:**

Behcet disease (BD) as a variable vessel vasculitis is mainly characterized by ocular involvement, genital and oral aphthosis, and erythema nodosum. However, major organ involvements including gastrointestinal involvement, nervous system, and vascular involvement are among the severe complications. Osteonecrosis is a rare complication of patients with BD. We aim to report the largest series of BD patients suffering from osteonecrosis.

**Methods:**

We have retrospectively reviewed all patients in Iran Behcet’s Disease Registry and reported those with osteonecrosis. Patients’ medication and clinical features, symptoms, and details of osteonecrosis will also be presented. Furthermore, previously reported cases will also be reviewed.

**Results:**

Seven thousand eight hundred thirty-one patients were diagnosed with BD and registered. 18 patients developed ON with an incidence of 0.22%. The most common involvement during the disease progression was oral aphthosis which appeared in 100% of patients followed by ocular involvement in 85.7% and skin involvement in 71.4%. Vascular, ocular, and nervous system involvements are significantly higher in BD patients with osteonecrosis than the other BD patients. For the management of acute episode of uveitis, deep vein thrombosis, severe gastrointestinal involvement, arterial involvement, nervous system Involvement, and joint involvement high dose of glucocorticoids is indicated.

**Conclusions:**

ON tends to appear as a multifocal involvement in BD patients, hence, after diagnosis of ON in one joint other possible sites of ON should be investigated.

**Supplementary Information:**

The online version contains supplementary material available at 10.1186/s41927-023-00366-3.

## Background

The initial symptoms of Behcet disease (BD) were first noticed by Hippocrates in ancient times [1]. In 1937, it was described as a separate disease by Huluci Behcet [2]. BD, first known as a viral syndrome, today is categorized as variable vessel vasculitis [1, 2]. Oral and genital aphthosis, erythema nodosum, arthritis, and ocular involvement are among the most common manifestation [1]. BD often follows a relapsing-remitting pattern; however, some of the organ involvement including the eye, vascular, gastrointestinal, and nervous system could result in severe and irreversible complications [3]. Osteonecrosis is a disturbing complication that has been frequently reported in lupus patients and is strongly associated with corticosteroid therapy. The pathologic pathway of corticosteroid-induced osteonecrosis is through suppression of both osteoblast and osteoclast and increasing apoptosis of osteoblast. Other suggested pathologic mechanisms of osteonecrosis include fat embolism, altered lipid metabolism, impaired repair of microfractures, primary cell death, vasculitis, and vasospasm [4]. Corticosteroid therapy is indicated in the major organ involvement and the flare of the disease [5]. The number of BD patients with osteonecrosis is uncommon in comparison with other rheumatologic diseases. Here we report the clinical features of 14 patients with BD who experienced osteonecrosis (ON). A literature review was also performed and previously published case reports are also discussed.

## Patients and methods

From 1975 to 2022 all patients diagnosed with BD were gathered and registered in Iran Behcet’s Disease Registry. The patients were diagnosed by expert opinion, even though most of them fulfilled one of the main classification criteria for BD. The patients’ demographic data in the data registry has already been discussed [6]. The diagnosis of ON was based on imaging (MRI and plain radiographs) and clinical symptoms and classified based on Ficat-Arlet staging. Patients’ medical records and charts were reviewed to look for demographic data, duration of follow-up and diagnosis, first manifestations, organ involvement, and lab test. Details of corticosteroid therapy and treatment regimen were also investigated.

## Search strategy

We have searched the Medline database from 9 May 1981 until 9 November 2022 to find manuscripts investigating osteonecrosis in patients with BD. The search strategy was built from combination of ‘Behcet’ and ‘osteonecrosis’ and related Mesh terms (Supplementary file 1). Case reports, randomized clinical trials, cohort, cross-sectional, case-control, and letters were included. Exclusion criteria were non-English manuscripts and missing data. After screening, one case series and six case reports were included [7–13]. Furthermore, reference lists of included manuscripts were searched by hand to ensure all relevant studies are included. In the final analysis 23 patients from 12 studies were included [7–18]. Summary of included studies are shown in Tables 1 and 2.

**Table 1:** Summary of previously reported and the present study BD patients with ON

**Author, year**	**Patient No.**	**Age (years), sex**	**Duration of the disease (years)**	**Time between diagnosis of BD and ON, (years)**	**Symptoms of BD before ON**	**Major organ involvement prior to ON**	**Site of ON**	**Relevant medication before ON**	**Treatment after diagnosis of ON**	**Lab test at the time of diagnosis of ON**
**Atas et al., 2019** [7]	#1	37, Male	13	8	na	Vascular and NS	Bilateral knee and hip	Azathioprine, Cyclophosphamide	Interferon	aCL IgG, IgM levels: Negative aB2-GPI IgM, IgG levels: Negative LDL:129 (mg/dl) TG:145 (mg/dl) Total Chol:203 (mg/dl) ESR:20 (mm/h) CRP:13 (mg/L)
	#2	37, Male	7	2	na	Vascular	Bilateral knee, left hip, left 3rd MCP	Azathioprine, Cyclophosphamide	Azathioprine	aCL IgG, IgM levels: Negative aB2-GPI IgM, IgG levels: Negative LDL:103 (mg/dl) TG:100 (mg/dl) Total Chol:163 (mg/dl) ESR:3 (mm/h) CRP:10 (mg/L)
	#3	62, Male	5	3	na	Vascular	Right hip	Azathioprine, Cyclophosphamide	Azathioprine	aCL IgG, IgM levels: Negative aB2-GPI IgM, IgG levels: Negative ESR:48 (mm/h) CRP:16 (mg/L)
	#4	19, Male	3	1	na	Ocular	Bilateral knee	Azathioprine, cyclosporine, Interferon, Infliximab	Infliximab	aCL IgG, IgM levels: Negative aB2-GPI IgM, IgG levels: Negative ESR:11 (mm/h) CRP:7 (mg/L)
	#5	39, Male	14	10	na	NS and Ocular	Bilateral knee	Azathioprine, Cyclosporine, Interferon, Infliximab Adalimumab	Adalimumab	aCL IgG, IgM levels: Negative aB2-GPI IgM, IgG levels: Negative LDL:83 (mg/dl) TG:185 (mg/dl) Total Chol: 156 (mg/dl) ESR:48 (mm/h) CRP:100 (mg/L)
	#6	43, Male	4	3	na	Vascular	Left hip	Azathioprine, Cyclophosphamide	Mycophenolate	aCL IgG, IgM levels: Negative aB2-GPI IgM, IgG levels: Negative ESR:33 (mm/h) CRP:7 (mg/L)
	#7	44, Male	17	16	na	Ocular	Bilateral hip	Azathioprine, Cyclosporine	Azathioprine	aCL IgG, IgM levels: 12, 11 aB2-GPI IgM, IgG levels: Negative ESR:76 (mm/h) CRP:100 (mg/L)
	#8	33, Male	14	14	na	Vascular	Bilateral knee	Azathioprine, Cyclophosphamide	Infliximab	aCL IgG, IgM levels: Negative aB2-GPI IgM, IgG levels: Negative LDL:204 (mg/dl) TG:127 (mg/dl) Total Chol:273 (mg/dl) ESR:9 (mm/h) CRP:3 (mg/L)
	#9	25, Male	4	1	na	Vascular	Bilateral knee	Interferon, Cyclophosphamide	Azathioprine	aCL IgG, IgM levels: 16, 18 aB2-GPI IgM, IgG levels: Negative ESR:32 (mm/h) CRP:57 (mg/L)
**Banicioiu-covei et al., 2019** [8]	#1	36, na	3 years after the initial symptoms	1 year after the initial symptoms	erythema nodosum, reduced visual acuity, oral aphthosis	Ocular, Skin	Right hip	Nonsteroidal anti-inflammatory drugs	na	ESR: 96 (mm/h) CRP:18.2 (mg/L) Leukocytes: 17,000/mm^3^ and 1 week later 24,500/mm^3^
**Chang et al., 2001** [10]	#1	45, Female	10 years after initial symptoms	10 years after initial symptoms	Recurrent oral ulcerations and genital ulcerations, recurrent erythema nodosum, positive pathergy test	Skin	Left knee	-	Colchicine, Aspirin, Nabumetone	Leukocytes: 10,200/mm^3^ Hematocrit: 39.3% Platelet: 370,000 Total protein: 6.1 (g/dl) Albumin: 3.6 (g/dl) AST: 14 (IU/l) ALT: 26 (IU/l) Creatinine: 0.8 (mg/dl) Cholesterol: 202 (mg/dl) Alkaline phosphatase: 117 (IU/l) ESR: 10 (mm/h) CRP: Negative Urinalysis: Normal Coagulation tests: Normal Rheumatoid factor: Negative Antinuclear antibody: Negative IgG aCL: 18 IgM aCL: Negative Lupus anticoagulant test: Negative HLA-B51: Positive
	#2	30, Male	5 years after the initial symptoms	2.5	Recurrent oral ulcerations, recurrent scrotal ulcerations, papulopustular eruptions, erythema nodosum-like lesions, posterior uveitis, seizure and multiple cerebral infarctions, intestinal hemorrhage, and multiple perforations of the cecum and ascending colon, pulmonary infarction	NS, Ocular, GI, and Respiratory system	Right hip	Azathioprine, Cyclosporine	na	WBC: 7100 Hematocrit: 38.9% Platelet: 202,000 Total protein: 6.6 (g/dl) Albumin: 3.9 (g/dl) AST: 12 (IU/l) ALT: 9 (IU/l) Creatinine: 1.0 (mg/dl) Cholesterol: 154 (mg/dl) Alkaline phosphatase: 124 (IU/l) ESR: 7 (mm/h) CRP: < 0.8 (mg/dl) Rheumatoid factor: Negative Antinuclear antibody: Negative Coagulation test: Normal Urinalysis: Normal HLA-B51: Positive IgM aCL antibody: 15 IgG aCL antibody: Negative Lupus anticoagulant: Negative
**Ersoz et al., 2013** [14]	#1	66, Female	10	10	oral aphthous ulcerations, erythema nodosum, uveitis, episcleritis, positive pathergy test, headaches, hemifacial spasms, and fever	NS	Right hip	Imuran, Colchicine, Amlodipine, and Prednisolone at exacerbations	na	CBC: Normal ESR: Normal CRP: Normal Liver function: Normal
**Essaadouni et al., 2017** [15]	#1	26, Female	6 years after the initial symptoms	3	oral ulcers (once a month) and recurring neurological manifestations consisting of headache, seizures and fever, pseudofolliculitis, positive pathergy test	NS	Unilateral hip	Colchicine, Azathioprine, Cyclophosphamide pulse	Tocilizumab	na
**Faezi et al., 2018** [16]	#1	26, Female	9 months after initial symptoms (until shoulder involvement) and 12 months until multifocal ON	4 months until shoulder involvement and 7 months until multifocal osteonecrosis	Oral aphtosis and decreased visual acuity (pan uveitis and retinal vasculitis)	Ocular	Right shoulder, bilateral knee, bilateral hip, right ankle	Azathioprine, Cyclophosphamide, Infliximab	na	aCL IgG, IgM: Normal Lupus anticoagulant: Normal aB2GP I IgG, IgM: Normal Coagulation tests: Normal Lipid profiles: Normal
**Lin et al., 2008** [17]	#1	49, Female	6	6	recurrent episodes of fever associated with oral and genital ulcers, positive pathergy test, abdominal pain, ileum perforation, diarrhea	GI	Bilateral Hip and left knee	na	Azathioprine	WBC: 13,900 with 68.3% neutrophils, 21.5% lymphocytes, and 9.2% monocytes Hemoglobin: 14.6 (g/dl) Platelet count 159,000
**Oktayoglu et al., 2012** [11]	#1	31, Female	12	12	Oral ulcerations, genital ulcerations, positive Pathergy test, posterior uveitis, pulmonary thromboembolism and thrombosis in superior vena cava, erythema nodosum like lesions, arthritis	Ocular, Skin, Vascular	Bilateral knee	Colchicine, Cyclophosphamide, Azathioprine, Nonsteroidal anti-inflammatory drugs	Indomethacin	WBC: 5250, Hematocrit: 36.3% Platelet: 383,000 Total protein: 8.3 (g/dL) Albumin: 4 (g/dL) AST: 15 (IU/L) ALT: 14 (IU/L) Creatinine: 0.58 (mg/dL) Alkaline phosphatase: 48 (IU/L) Parathyroid hormone: 36.82 (pg/mL) ESR: 46 (mm/hour) CRP: 0.58 (mg/dL) Thyroid hormone levels: Normal Urinalysis: Normal Coagulation tests: Normal Protein C and Protein S values: Normal aCL IgG and IgM: Negative Rheumatoid factor: Negative Antinuclear antibody: Negative anti-dsDNA: Negative
**Polat et al., 2010** [9]	#1	38, Female	5 years after the initial symptoms	7 months	Recurrent oral ulcers with 5-year duration and genital ulcers persisting for 1 year, acute pan uveitis (left eye), chronic uveitis (right eye), positive pathergy test	Ocular	Bilateral knee	Azathioprine, Colchicine, Warfarin, Acetazolamide	na	CRP: 68 (mg/dl) ESR: 60 (mm/h) The other hematological and biochemical tests are normal aCL IgM and IgG antibodies: Negative
**Vaiopoulos et al., 2019** [18]	Aseptic bone necrosis was seen in 3 male patients (left hip, right hip, and both hips)									
**Varoglu et al., 2017** [13]	#1	36, Male	1	1	Oral aphthous ulcerations, gait disorder, diplopia, and speech disorder, genital aphthous ulcerations	NS	Bilateral hip	-	na	HLA B51: Positive The markers of collagen tissue disease: Negative Hematological markers (fibrinogen, antithrombin, and protein C and S): Normal Factor V Leiden, prothrombin G20210A gene mutations, and MTHFR enzyme: Negative The plasma Vit B (12), folic acid, and homocysteine levels: Normal
**Yapar et al., 2001** [12]	#1	35, Male	na	na	Uveitis	Ocular	Bilateral knee	Cyclosporine A, Nonsteroidal anti-inflammatory drugs	na	Na
**The present study**	#1	37, Male	4	15 months	Oral aphthosis, Genital aphthosis, pseudofolliculitis, pan ophthalmitis, GI aphthosis, CNS vasculitis	Ocular, Skin, NS, GI	Left Hip	Azathioprine, Cyclophosphamide, Alendronate	na	Pathergy test: Negative HLA B51: Positive ESR: High Hyperlipidemia: Negative
	#2	25, Male	4	2	Oral aphthosis, Erythema Nodosum, pan ophthalmitis, DVT, oligoarthritis	Skin, Ocular, Vascular, Musculoskeletal	2 Hips 2 Shoulders	Azathioprine, Methotrexate, Cyclophosphamide, Infliximab	na	Pathergy test: Negative ESR: High HLA B51: Negative Hyperlipidemia: Positive Fatty liver: Negative Osteoporosis: Positive
	#3	28, Male	9	1	Oral aphthosis, Genital aphthosis, Erythema Nodosum, aneurysm, monoarthritis, Epididymitis,	Skin, Vascular, Musculoskeletal	Left Hip	Azathioprine, Cyclophosphamide, Anticoagulant	na	Pathergy test: Negative HLA B51: Negative ESR: High Hyperlipidemia: Negative Fatty liver: Negative Cushingoid: Negative
	#4	28, Male	6	7 months	Oral aphthosis, Genital aphthosis, Skin Aphthosis, pan ophthalmitis, monoarthritis	Skin, Ocular, Musculoskeletal	Right Hip	Azathioprine, Cyclophosphamide, Alendronate	na	Pathergy test: Positive HLA B51: Negative ESR: High Antiphospholipid antibodies: Negative Hyperlipidemia: Negative Fatty liver: Negative Cushingoid: Negative
	#5	19, Male	10	6 months	Oral aphthosis, Genital aphthosis, pan uveitis, DVT	Ocular, Vascular	Bilateral hip	Methotrexate, Alendronate	na	Pathergy test: Positive HLA B51: Negative ESR: less than 10mm/h Antiphospholipid antibodies: Negative Hyperlipidemia: Negative Fatty liver: Negative Cushingoid: Negative
	#6	36, Male	12	8	Uveitis, Oral aphthosis, pseudo folliculitis, pan ophthalmitis	Ocular, Skin	Right Hip	Azathioprine, Methotrexate, Alendronate	na	Pathergy test: Negative HLA B51: Positive ESR: High Antiphospholipid antibodies: Negative Hyperlipidemia: Positive
	#7	38, Male	12	8	Oral aphthosis, Genital aphthosis, pan uveitis, arthralgia	Ocular, Musculoskeletal	Bilateral hip	Methotrexate, Alendronate	na	Pathergy test: Positive HLA B51: Positive ESR: less than 10mm/h Antiphospholipid antibodies: Negative Hyperlipidemia: Negative
	#8	17, Female	5	1	Oral aphthosis, pan ophthalmitis, oligoarthritis	Ocular, Musculoskeletal	Bilateral hip	Azathioprine, Cyclophosphamide, Alendronate	na	Pathergy test: Positive HLA B51: Negative ESR: less than 10mm/h Antiphospholipid antibodies: Negative Hyperlipidemia: Negative Osteoporosis: Negative
	#9	17, Male	7	19 months	Oral aphthosis, Pseudo Folliculitis, aneurysm, CNS vasculitis	Skin, Vascular, NS	Bilateral hip	Methotrexate, Cyclophosphamide, Alendronate, Aspirin	na	Pathergy test: Positive HLA B51: Negative ESR: less than 10mm/h Antiphospholipid antibodies: Negative Hyperlipidemia: Negative Osteoporosis: Positive Cushingoid: Positive
	#10	31, Male	8	20 months	Oral aphthosis, Genital aphthosis, Pseudo Folliculitis, pan ophthalmitis	Skin, Ocular	Bilateral hip	Methotrexate, Alendronate	na	Pathergy test: Positive HLA B51: Negative ESR: less than 10mm/h Hyperlipidemia: Negative Osteoporosis: Positive
	#11	36, Male	8	1	Oral aphthosis, pan ophthalmitis, arthralgia, Epididymitis, CNS vasculitis	Ocular, Musculoskeletal, NS	Right Hip	Azathioprine, Cyclophosphamide, Alendronate	na	Pathergy test: Negative HLA B51: Negative ESR: High Hyperlipidemia: Negative Fatty liver: Negative Osteoporosis: Positive
	#12	24, Male	6	40 months	Oral aphthosis, Pseudo folliculitis& Erythema Nodosum, pan uveitis, CNS vasculitis	Skin, Ocular, NS	Bilateral hip	Methotrexate, Cyclophosphamide	na	Pathergy test: Positive HLA B51: Positive ESR: High Antiphospholipid antibodies: Negative Hyperlipidemia: Negative Fatty liver: Negative Osteoporosis: Positive Cushingoid: Negative
	#13	20, Male	6	8 months	Oral aphthosis, Genital aphthosis, Erythema Nodosum, pan ophthalmitis, pulmonary Aneurysm,	Skin, Ocular, Vascular	Bilateral hip	Azathioprine, Anticoagulant	na	Pathergy test: Negative HLA B51: Negative ESR: less than 10mm/h Antiphospholipid antibodies: Negative Hyperlipidemia: Negative Fatty liver: Negative Osteoporosis: Positive Cushingoid: Negative
	#14	39.5 Male	15	9.5	Oral aphthosis, Pseudo folliculitis, pan ophthalmitis,	Skin, Ocular	Bilateral hip	Methotrexate	na	Pathergy test: Negative HLA B51: Positive ESR: less than 10mm/h Antiphospholipid antibodies: Negative Hyperlipidemia: Negative Fatty liver: Negative Osteoporosis: Positive Cushingoid: Positive

*BD* Behcet disease, *ON* Osteonecrosis, *aCL* Anti-cardiolipin, *aB2-GPI* Anti-B2 glycoprotein I, *NS* Nervous system, *LDL* Low-density lipoprotein, *TG* Triglyceride, *T. Chol* Total cholesterol, *ESR* Erythrocyte sedimentation rate, *CRP* C-reactive protein, *na* Not applicable, *MCP* Metacarpophalangeal joint, *AST* Aspartate transaminase, *ALT* Alanine transaminase, *HLA B51* Human leukocyte antigen 51, *GI* Gastrointestinal, *WBC* White blood cell, *CBC* Complete blood cell, *DVT* Deep vein thrombosis, *CNS* Central nervous system

**Table 2:** Summary of corticosteroid therapy BD patients with ON

**Author, year**	**Patients No.**	**Highest daily corticosteroid dose before ON (mg/day)**	**Number of corticosteroid pulses before ON**	**Time between initial corticosteroid dose and ON (months)**	**Cumulative corticosteroid dose prior to ON (g)**
**Atas et al., 2019** [7]	#1	64	1000 mg/month, 8 cycles	24	18
	#2	80	1000 mg/month, 8 cycles	30	18.4
	#3	48	500 mg/q2w, 8 cycles	27	14.2
	#4	80	Single 500 mg infusion	20	6
	#5	60	1000 mg/month, 5 cycles	30	11
	#6	64	1000 mg for 3 consecutive days/2 cycles	24	21
	#7	60	1000 mg/day, 3 cycles	100	19
	#8	64	1000 mg for 7 consecutive days	2	10
	#9	64	1000 mg/month, 8 cycles	24	18
**Banicioiu-covei et al., 2019** [8]	1	na	na	Less than 12 months	na
**Chang et al., 2001** [10]	#1	0	0	0	0
	#2	na	na	36	na
**Ersoz et al., 2013** [14]	1	na	na	na	na
**Essaadouni et al., 2017** [15]	1	na	na	36	na
**Faezi et al., 2018** [16]	1	na	0	4	na
**Lin et al., 2008** [17]	1	na	na	na	na
**Oktayoglu et al., 2012** [11]	1	na	na	120	na
**Polat et al., 2010** [9]	1	na	0	7	na
**Vaiopoulos et al., 2019** [18]	#1 & #2 & #3	na	na	na	na
**Varoglu et al., 2017** [13]	1	na	methylprednisolone [5-day 1000 mg/day intravenous (IV) infusion]	12	na
**Yapar et al., 2001** [12]	1	na	na	na	na
**The present study**	#1	60	5	15	15.24
	#2	45	1	28	-20.02 g before the first AVN -22.26 g before the second AVN
	#3	30	0	12	5.74
	#4	30	0	7	5.88
	#5	30	0	6	5.04
	#6	30	0	90	17.57
	#7	30	0	24	3.56
	#8	75	0	11	5.81
	#9	60	0	19	17.57
	#10	30	0	12	5.42
	#11	30	0	12	7.56
	#12	30	5	40	22.92
	#13	40	0	8	7.28
	#14	40	0	108	45.43

*ON* Osteonecrosis

## Statistical analysis

Statistical analysis was conducted using SPSS version 23 for windows (IBM, Armonk, New York). Continuous variables are reported as mean ± sd. Categorical variables are reported as numbers and percentages.

## Results

### Demographic data and clinical manifestations

Seven thousand eight hundred thirty-one patients were diagnosed with BD and registered. The incidence of ON in our dataset is 0.22%. All of them fulfilled the International Criteria for Behcet’s Disease (ICBD) classification criteria for BD. Three patients were lost to follow up. One patient with multifocal osteonecrosis has already been reported [16]. 14 patients remained for further report including 13 males and one female. The mean age at the onset of the disease was 25.14 ± 6.64 years (range 15–36). The patients’ demographic data are shown in Table 1.

The first manifestation was oral aphthosis in all patients except one who presented with uveitis. The most common involvement during the disease progression was oral aphthosis which appeared in 100% of patients followed by ocular involvement in 85.71% and skin involvement in 71.43%. In those with skin and ocular involvement, pseudofolliculitis and pan ophthalmitis were prevalent, respectively.

HLA-B51 was presented in 35.71% of patients. In none of the cases, the antiphospholipid antibody was detected and the result of the pathergy skin test was positive in 50% of patients.

### Corticosteroid therapy

The mean cumulative dosage of corticosteroid therapy received by patients before ON is 13,377.14 ± 11,462.30 mg (range 3,560–45,430). The highest daily dosage of corticosteroid therapy ranged from 30 to 75 mg. Table 2 demonstrates details of corticosteroid therapy. Osteoporosis, cushingoid condition, and hyperlipidemia were investigated as the complications of corticosteroid therapy. Osteoporosis as the most common complication occurred in 87.5% of patients.

### ON

Twenty-five joints were involved in 14 patients. In 13 patients, the hip was the only joint complicated with ON and in one patient ON occurred in both shoulders and both hips. Nine patients developed bilateral hip osteonecrosis. Eleven patients were diagnosed with ON at stage III of the disease based on Ficat-Arlet staging. The mean duration between the first symptoms of BD and pain in the involved joint was 89.29 ± 36.96 (range 41–162) months. However, BD and ON are diagnosed with a delay of 74.21 ± 35.97 months and 6.71 ± 5.57 months respectively. This results in a span of 34.36 ± 37.87 months between the diagnosis of BD and the diagnosis of ON. The mean Duration of steroid therapy before development of osteonecrosis was 28 ± 31.6 months. No symptoms of arthritis were detected before the ON in the involved joints (Table 3).

**Table 3:** Patients’ data pertained to osteonecrosis

	**Patient No 1**	**Patient No 2**	**Patient No 3**	**Patient No 4**	**Patient No 5**	**Patient No 6**	**Patient No 7**	**Patient No 8**	**Patient No 9**	**Patient No 10**	**Patient No 11**	**Patient No 12**	**Patient No 13**	**Patient No 14**
**Number of joints involved by ON**	1	4	1	1	2	1	2	2	2	2	1	2	2	2
**Site of ON**	Left Hip	2 Hips 2 Shoulders	Left Hip	Right Hip	2 Hips	Right Hip	2 Hips	2 Hips	2 Hips	2 Hips	Right Hip	2 Hips	2 Hips	2 Hips
**Stage of ON in diagnosis time**	III	-II/III for hips -III/IV for shoulders	II	III	III	III	III	II	III	III	III	III	II	III/IV
**The duration between joint pain to the diagnosis of ON (delayed diagnosis of ON)**	7 months	5 months for the first AVN 18 months for the second AVN	2 months	2 months	3 months	16 months	7 months	3 months	8 months	3 months	0	14 months	6 months	18 months
**The duration between the first and second ON**	-	17 months	-	-	-	-	-	-	-	-	-	-	-	-
**The duration between vascular involvement to ON**	-	3 years	5 years	-	2 years	No	No	No	19 months	No	No	No	8 months	No
**Previous arthritis at the site of ON**	No	No	No	No	No	No	No	N0	No	No	No	No	No	No

*BD* Behcet patients, *ON* Osteonecrosis

### Medication

Among immunosuppressant drugs in the management of BD azathioprine, methotrexate, and cyclophosphamide pulse were used in 57.14% of patients each. Alendronate was used in 64.28% of patients (Table 1).

## Discussion

In comparison with the previously published case series, we have reported the highest number of BD patients with ON. The mean age of all patients (including our dataset and the literature) is 33.08 years. Of the 36 patients (one study didn’t provide information regarding patient’s sex) eight patients are female.

As the cumulative dose of exogenous corticosteroids increases, the probability of osteonecrosis increases dramatically. This could be due to prolonged exposure or pulses [19]. The cumulative dosage of corticosteroids exceeds 15 gr in six of 14 patients in our dataset. Three of them were because of pulse therapy and two were exposed to corticosteroids for more than 7 years. The last one had the second-highest dosage of corticosteroid usage per day. Cumulative dosage of corticosteroids was more than 15gr in five of the ten patients in the literature. However, in the study by Chang et al. a patient developed ON and no history of corticosteroid therapy was reported. In our dataset three patients required corticosteroid pulses while in the literature 10 of the 13 patients had corticosteroid pulses before development of ON. Patients develop ON on an average of 30.62 months after first exposure to corticosteroids. The odds ratio of developing osteonecrosis in patients with high cumulative dosage of corticosteroid therapy (more than 10g) is 2.4 (95% CI. 0.8 to 6.4) and osteonecrosis occur in 6.7% of patients with high dosage of corticosteroid therapy [20]. Although 50 percent of BD patients experience ocular involvement, and corticosteroid therapy is a common regimen specifically in the flare of the disease the incidence ON in BD patients remains low [5].

In our case series proportion of ocular (85.7%), vascular (35.7%), and neurologic (28.6%) involvements are significantly higher than other patients with BD [1]. In the Iran data registry, the incidence of ocular, vascular, and neurologic involvements are 56.8%, 8.3%, and 3.7% respectively [1]. Osteonecrosis could be due to the high dosage of corticosteroids which is required to overcome these complications [5]. In the literature the vascular (30.43%), Ocular (34.78%), and NS (26.1%) were the main organ involvements. Vascular involvement in BD may also play a role in progression of ON, however, there is not enough evidence to support this hypothesis [21]. In the study by Elgengehy et al. Vasculitis damage index in BD patients is significantly associated with ON [22]. Vascular damage and impaired endothelial dysfunction play different role in development of ON [23]. So cumulative effect of vasculitis and high dose corticosteroid in these patients may be considerable in ON occurrence. In the literature 26.1% of patients experienced NS involvement. In our dataset 28.57% of patients suffered from NS involvement, while, the proportion of patients with NS involvement in Iran data registry is approximately 3% and in Germany Registry, Turkey Cohort, ICBD 27 countries, and Inssbruck Cohort is lower than 24% [1]. In a cohort study in 2021 nervous system (NS) involvement was the independent risk factors of osteonecrosis in lupus patients [24].

The most common site of osteonecrosis was hip involvement which occurred in 27 of the 37 patients. In the literature 26.08% of patients had bilateral hip involvement and 43.48% of patients suffered from bilateral knee ON. The incidence of unilateral hip involvement in the literature is 39.13%. In our dataset, all patients had ON of the hip joint and in 64.28% of cases, the ON appeared as a bilateral involvement [7]. In patients with secondary induced osteonecrosis, 64% of cases with atraumatic osteonecrosis of the knee and 55% of those with atraumatic osteonecrosis of the hip joint are bilateral cases [25, 26]. Based on Ficat-Arlet classification 71.43% of our patients diagnosed with stage III of osteonecrosis of the hip joint which is comparable with cases suffering from secondary induced osteonecrosis of the hip joint [25]. Mont et al. reported a weighted mean time of 49 months (range two-143 months) from diagnosis of asymptomatic disease to collapse of the femoral head [27]. Furthermore more than 50% of early-stage osteonecrosis of the hip and 96% of the stage III of osteonecrosis of the hip will progress to collapse of the hip joint [27].

## Limitation and strengths

Since the number of BD patients with ON is very low in comparison with whole data registry, we were not able to conduct an analysis due to random error. Also, three patients were lost to follow-up which is a considerable number in comparison with our data set (14 patients). However, our study is the largest reported case series of BD patients with ON and almost every clinical feature of patients and ON are reported and discussed. The mechanism of ON in BD patients is not completely understood and further longitudinal studies are needed to prove the role of corticosteroids and vascular complications or other risk factors in the development of ON in BD patients.

## Conclusion

Corticosteroid therapy is the main risk factor of ON in BD patients. ON tend to appear as a multifocal involvement in BD patient, hence, after diagnosis of ON in one joint other possible sites of ON should be investigated. Vascular involvement is one of the most prevalent major organ complications in BD patients. The mechanism of vascular involvement in osteonecrosis of BD patients and the cumulative effect of corticosteroid therapy should be clarified in the future.

### Supplementary Information

**Additional file 1.** Search strategy consisted of ‘Behcet disease’ OR related mesh terms AND ‘Osteonecrosis’ OR related mesh terms.

## Data Availability

All data generated or analysed during this study are included in this published article.

## Abbreviations

BD: Behcet disease
ON: Osteonecrosis
aCL: Anti-cardiolipin
aB2-GPI: Anti-B2 glycoprotein I
NS: Nervous system
ESR: Erythrocyte sedimentation rate
CRP: C-reactive protein
HLA B5: Human leukocyte antigen 5
GI: Gastrointestinal
WBC: White blood cell
