# Supplementary material for: Osteonecrosis as a rare musculoskeletal complication in Behcet’s disease- the largest case series with literature review
Source: BMC Rheumatol. 2023 Nov 30;7:42. doi: 10.1186/s41927-023-00366-3 (PMC10687826; doi:10.1186/s41927-023-00366-3)
Supplement: Supplementary file 1 — Additional file 1. Search strategy consisted of ‘Behcet disease’ OR related mesh terms AND ‘Osteonecrosis’ OR related mesh terms. [file 41927_2023_366_MOESM1_ESM.docx]

| **Search strategy** | **Results** |
| --- | --- |
| ("Osteonecrosis"[MeSH Terms] OR ("Osteonecrosis"[MeSH Terms] OR "Osteonecrosis"[All Fields] OR "osteonecroses"[All Fields]) OR ("Osteonecrosis"[MeSH Terms] OR "Osteonecrosis"[All Fields] OR "osteonecroses"[All Fields]) OR ("Osteonecrosis"[MeSH Terms] OR "Osteonecrosis"[All Fields] OR ("bone"[All Fields] AND "necrosis"[All Fields]) OR "bone necrosis"[All Fields]) OR ("Osteonecrosis"[MeSH Terms] OR "Osteonecrosis"[All Fields] OR ("bone"[All Fields] AND "necroses"[All Fields]) OR "bone necroses"[All Fields]) OR ("Osteonecrosis"[MeSH Terms] OR "Osteonecrosis"[All Fields] OR ("necroses"[All Fields] AND "bone"[All Fields]) OR "necroses bone"[All Fields]) OR ("Osteonecrosis"[MeSH Terms] OR "Osteonecrosis"[All Fields] OR ("necrosis"[All Fields] AND "bone"[All Fields]) OR "necrosis bone"[All Fields]) OR ("Osteonecrosis"[MeSH Terms] OR "Osteonecrosis"[All Fields] OR ("necrosis"[All Fields] AND "avascular"[All Fields] AND "bone"[All Fields])) OR ("Osteonecrosis"[MeSH Terms] OR "Osteonecrosis"[All Fields] OR ("avascular"[All Fields] AND "necrosis"[All Fields] AND "bone"[All Fields]) OR "avascular necrosis of bone"[All Fields]) OR ("Osteonecrosis"[MeSH Terms] OR "Osteonecrosis"[All Fields] OR ("bone"[All Fields] AND "avascular"[All Fields] AND "necrosis"[All Fields]) OR "bone avascular necrosis"[All Fields]) OR ("Osteonecrosis"[MeSH Terms] OR "Osteonecrosis"[All Fields] OR ("kienbock"[All Fields] AND "disease"[All Fields]) OR "kienbock disease"[All Fields]) OR ("Osteonecrosis"[MeSH Terms] OR "Osteonecrosis"[All Fields] OR ("kienbock s"[All Fields] AND "disease"[All Fields]) OR "kienbock s disease"[All Fields]) OR ("Osteonecrosis"[MeSH Terms] OR "Osteonecrosis"[All Fields] OR ("kienboeck"[All Fields] AND "disease"[All Fields]) OR "kienboeck disease"[All Fields]) OR ("Osteonecrosis"[MeSH Terms] OR "Osteonecrosis"[All Fields] OR ("kienboeck s"[All Fields] AND "disease"[All Fields]) OR "kienboeck s disease"[All Fields]) OR ("Osteonecrosis"[MeSH Terms] OR "Osteonecrosis"[All Fields] OR ("kienboecks"[All Fields] AND "disease"[All Fields]) OR "kienboecks disease"[All Fields]) OR ("Osteonecrosis"[MeSH Terms] OR "Osteonecrosis"[All Fields] OR ("necrosis"[All Fields] AND "aseptic"[All Fields] AND "bone"[All Fields])) OR ("Osteonecrosis"[MeSH Terms] OR "Osteonecrosis"[All Fields] OR ("aseptic"[All Fields] AND "necrosis"[All Fields] AND "bone"[All Fields]) OR "aseptic necrosis of bone"[All Fields]) OR ("Osteonecrosis"[MeSH Terms] OR "Osteonecrosis"[All Fields] OR ("bone"[All Fields] AND "aseptic"[All Fields] AND "necrosis"[All Fields]) OR "bone aseptic necrosis"[All Fields])) AND ("Behcet Syndrome"[MeSH Terms] OR ("Behcet Syndrome"[MeSH Terms] OR ("behcet"[All Fields] AND "syndrome"[All Fields]) OR "Behcet Syndrome"[All Fields]) OR ("Behcet Syndrome"[MeSH Terms] OR ("behcet"[All Fields] AND "syndrome"[All Fields]) OR "Behcet Syndrome"[All Fields] OR ("behcet s"[All Fields] AND "syndrome"[All Fields]) OR "behcet s syndrome"[All Fields]) OR ("Behcet Syndrome"[MeSH Terms] OR ("behcet"[All Fields] AND "syndrome"[All Fields]) OR "Behcet Syndrome"[All Fields] OR ("triple"[All Fields] AND "symptom"[All Fields] AND "complex"[All Fields]) OR "triple symptom complex"[All Fields]) OR ("Behcet Syndrome"[MeSH Terms] OR ("behcet"[All Fields] AND "syndrome"[All Fields]) OR "Behcet Syndrome"[All Fields] OR ("triple"[All Fields] AND "symptom"[All Fields] AND "complex"[All Fields]) OR "triple symptom complex"[All Fields]) OR ("Behcet Syndrome"[MeSH Terms] OR ("behcet"[All Fields] AND "syndrome"[All Fields]) OR "Behcet Syndrome"[All Fields] OR ("symptom"[All Fields] AND "complex"[All Fields] AND "triple"[All Fields])) OR ("Behcet Syndrome"[MeSH Terms] OR ("behcet"[All Fields] AND "syndrome"[All Fields]) OR "Behcet Syndrome"[All Fields] OR ("triple"[All Fields] AND "symptom"[All Fields] AND "complices"[All Fields])) OR ("Behcet Syndrome"[MeSH Terms] OR ("behcet"[All Fields] AND "syndrome"[All Fields]) OR "Behcet Syndrome"[All Fields] OR ("behcet"[All Fields] AND "disease"[All Fields]) OR "behcet disease"[All Fields]) OR ("Behcet Syndrome"[MeSH Terms] OR ("behcet"[All Fields] AND "syndrome"[All Fields]) OR "Behcet Syndrome"[All Fields] OR ("behcet"[All Fields] AND "diseases"[All Fields]) OR "behcet diseases"[All Fields]) OR ("Behcet Syndrome"[MeSH Terms] OR ("behcet"[All Fields] AND "syndrome"[All Fields]) OR "Behcet Syndrome"[All Fields] OR ("adamantiades"[All Fields] AND "behcet"[All Fields] AND "disease"[All Fields]) OR "adamantiades behcet disease"[All Fields]) OR ("Behcet Syndrome"[MeSH Terms] OR ("behcet"[All Fields] AND "syndrome"[All Fields]) OR "Behcet Syndrome"[All Fields] OR ("adamantiades"[All Fields] AND "behcet"[All Fields] AND "disease"[All Fields]) OR "adamantiades behcet disease"[All Fields]) OR ("Behcet Syndrome"[MeSH Terms] OR ("behcet"[All Fields] AND "syndrome"[All Fields]) OR "Behcet Syndrome"[All Fields] OR ("adamantiades"[All Fields] AND "behcet"[All Fields] AND "diseases"[All Fields])) OR ("Behcet Syndrome"[MeSH Terms] OR ("behcet"[All Fields] AND "syndrome"[All Fields]) OR "Behcet Syndrome"[All Fields] OR ("behcet"[All Fields] AND "triple"[All Fields] AND "symptom"[All Fields] AND "complex"[All Fields])) OR ("Behcet Syndrome"[MeSH Terms] OR ("behcet"[All Fields] AND "syndrome"[All Fields]) OR "Behcet Syndrome"[All Fields] OR ("old"[All Fields] AND "silk"[All Fields] AND "route"[All Fields] AND "disease"[All Fields]) OR "old silk route disease"[All Fields]) OR ("Behcet Syndrome"[MeSH Terms] OR ("behcet"[All Fields] AND "syndrome"[All Fields]) OR "Behcet Syndrome"[All Fields] OR ("behcet s"[All Fields] AND "disease"[All Fields]) OR "behcet s disease"[All Fields]) OR ("Behcet Syndrome"[MeSH Terms] OR ("behcet"[All Fields] AND "syndrome"[All Fields]) OR "Behcet Syndrome"[All Fields] OR ("behcet"[All Fields] AND "disease"[All Fields]) OR "behcet disease"[All Fields]))  Translations  Osteonecrosis: "osteonecrosis"[MeSH Terms] OR "osteonecrosis"[All Fields] OR "osteonecroses"[All Fields]  Osteonecroses: "osteonecrosis"[MeSH Terms] OR "osteonecrosis"[All Fields] OR "osteonecroses"[All Fields]  Bone Necrosis: "osteonecrosis"[MeSH Terms] OR "osteonecrosis"[All Fields] OR ("bone"[All Fields] AND "necrosis"[All Fields]) OR "bone necrosis"[All Fields]  Bone Necroses: "osteonecrosis"[MeSH Terms] OR "osteonecrosis"[All Fields] OR ("bone"[All Fields] AND "necroses"[All Fields]) OR "bone necroses"[All Fields]  Necroses, Bone: "osteonecrosis"[MeSH Terms] OR "osteonecrosis"[All Fields] OR ("necroses"[All Fields] AND "bone"[All Fields]) OR "necroses, bone"[All Fields]  Necrosis, Bone: "osteonecrosis"[MeSH Terms] OR "osteonecrosis"[All Fields] OR ("necrosis"[All Fields] AND "bone"[All Fields]) OR "necrosis bone"[All Fields]  Necrosis, Avascular, of Bone: "osteonecrosis"[MeSH Terms] OR "osteonecrosis"[All Fields] OR ("necrosis"[All Fields] AND "avascular"[All Fields] AND "bone"[All Fields]) OR "necrosis, avascular, of bone"[All Fields]  Avascular Necrosis of Bone: "osteonecrosis"[MeSH Terms] OR "osteonecrosis"[All Fields] OR ("avascular"[All Fields] AND "necrosis"[All Fields] AND "bone"[All Fields]) OR "avascular necrosis of bone"[All Fields]  Bone Avascular Necrosis: "osteonecrosis"[MeSH Terms] OR "osteonecrosis"[All Fields] OR ("bone"[All Fields] AND "avascular"[All Fields] AND "necrosis"[All Fields]) OR "bone avascular necrosis"[All Fields]  Kienbock Disease: "osteonecrosis"[MeSH Terms] OR "osteonecrosis"[All Fields] OR ("kienbock"[All Fields] AND "disease"[All Fields]) OR "kienbock disease"[All Fields]  Kienbock's Disease: "osteonecrosis"[MeSH Terms] OR "osteonecrosis"[All Fields] OR ("kienbock's"[All Fields] AND "disease"[All Fields]) OR "kienbock's disease"[All Fields]  Kienboeck Disease: "osteonecrosis"[MeSH Terms] OR "osteonecrosis"[All Fields] OR ("kienboeck"[All Fields] AND "disease"[All Fields]) OR "kienboeck disease"[All Fields]  Kienboeck's Disease: "osteonecrosis"[MeSH Terms] OR "osteonecrosis"[All Fields] OR ("kienboeck's"[All Fields] AND "disease"[All Fields]) OR "kienboeck's disease"[All Fields]  Kienboecks Disease: "osteonecrosis"[MeSH Terms] OR "osteonecrosis"[All Fields] OR ("kienboecks"[All Fields] AND "disease"[All Fields]) OR "kienboecks disease"[All Fields]  Necrosis, Aseptic, of Bone: "osteonecrosis"[MeSH Terms] OR "osteonecrosis"[All Fields] OR ("necrosis"[All Fields] AND "aseptic"[All Fields] AND "bone"[All Fields]) OR "necrosis, aseptic, of bone"[All Fields]  Aseptic Necrosis of Bone: "osteonecrosis"[MeSH Terms] OR "osteonecrosis"[All Fields] OR ("aseptic"[All Fields] AND "necrosis"[All Fields] AND "bone"[All Fields]) OR "aseptic necrosis of bone"[All Fields]  Bone Aseptic Necrosis: "osteonecrosis"[MeSH Terms] OR "osteonecrosis"[All Fields] OR ("bone"[All Fields] AND "aseptic"[All Fields] AND "necrosis"[All Fields]) OR "bone aseptic necrosis"[All Fields]  Behcet Syndrome: "behcet syndrome"[MeSH Terms] OR ("behcet"[All Fields] AND "syndrome"[All Fields]) OR "behcet syndrome"[All Fields]  Behcet's Syndrome: "behcet syndrome"[MeSH Terms] OR ("behcet"[All Fields] AND "syndrome"[All Fields]) OR "behcet syndrome"[All Fields] OR ("behcet's"[All Fields] AND "syndrome"[All Fields]) OR "behcet's syndrome"[All Fields]  Triple-Symptom Complex: "behcet syndrome"[MeSH Terms] OR ("behcet"[All Fields] AND "syndrome"[All Fields]) OR "behcet syndrome"[All Fields] OR ("triple"[All Fields] AND "symptom"[All Fields] AND "complex"[All Fields]) OR "triple symptom complex"[All Fields]  Triple Symptom Complex: "behcet syndrome"[MeSH Terms] OR ("behcet"[All Fields] AND "syndrome"[All Fields]) OR "behcet syndrome"[All Fields] OR ("triple"[All Fields] AND "symptom"[All Fields] AND "complex"[All Fields]) OR "triple symptom complex"[All Fields]  Symptom Complex, Triple: "behcet syndrome"[MeSH Terms] OR ("behcet"[All Fields] AND "syndrome"[All Fields]) OR "behcet syndrome"[All Fields] OR ("symptom"[All Fields] AND "complex"[All Fields] AND "triple"[All Fields]) OR "symptom complex, triple"[All Fields]  Triple Symptom Complices: "behcet syndrome"[MeSH Terms] OR ("behcet"[All Fields] AND "syndrome"[All Fields]) OR "behcet syndrome"[All Fields] OR ("triple"[All Fields] AND "symptom"[All Fields] AND "complices"[All Fields]) OR "triple symptom complices"[All Fields]  Behcet Disease: "behcet syndrome"[MeSH Terms] OR ("behcet"[All Fields] AND "syndrome"[All Fields]) OR "behcet syndrome"[All Fields] OR ("behcet"[All Fields] AND "disease"[All Fields]) OR "behcet disease"[All Fields]  Behcet Diseases: "behcet syndrome"[MeSH Terms] OR ("behcet"[All Fields] AND "syndrome"[All Fields]) OR "behcet syndrome"[All Fields] OR ("behcet"[All Fields] AND "diseases"[All Fields]) OR "behcet diseases"[All Fields]  Adamantiades-Behcet Disease: "behcet syndrome"[MeSH Terms] OR ("behcet"[All Fields] AND "syndrome"[All Fields]) OR "behcet syndrome"[All Fields] OR ("adamantiades"[All Fields] AND "behcet"[All Fields] AND "disease"[All Fields]) OR "adamantiades behcet disease"[All Fields]  Adamantiades Behcet Disease: "behcet syndrome"[MeSH Terms] OR ("behcet"[All Fields] AND "syndrome"[All Fields]) OR "behcet syndrome"[All Fields] OR ("adamantiades"[All Fields] AND "behcet"[All Fields] AND "disease"[All Fields]) OR "adamantiades behcet disease"[All Fields]  Adamantiades-Behcet Diseases: "behcet syndrome"[MeSH Terms] OR ("behcet"[All Fields] AND "syndrome"[All Fields]) OR "behcet syndrome"[All Fields] OR ("adamantiades"[All Fields] AND "behcet"[All Fields] AND "diseases"[All Fields]) OR "adamantiades behcet diseases"[All Fields]  Behcet Triple Symptom Complex: "behcet syndrome"[MeSH Terms] OR ("behcet"[All Fields] AND "syndrome"[All Fields]) OR "behcet syndrome"[All Fields] OR ("behcet"[All Fields] AND "triple"[All Fields] AND "symptom"[All Fields] AND "complex"[All Fields]) OR "behcet triple symptom complex"[All Fields]  Old Silk Route Disease: "behcet syndrome"[MeSH Terms] OR ("behcet"[All Fields] AND "syndrome"[All Fields]) OR "behcet syndrome"[All Fields] OR ("old"[All Fields] AND "silk"[All Fields] AND "route"[All Fields] AND "disease"[All Fields]) OR "old silk route disease"[All Fields]  Behcet's Disease: "behcet syndrome"[MeSH Terms] OR ("behcet"[All Fields] AND "syndrome"[All Fields]) OR "behcet syndrome"[All Fields] OR ("behcet's"[All Fields] AND "disease"[All Fields]) OR "behcet's disease"[All Fields]  Behcet Disease: "behcet syndrome"[MeSH Terms] OR ("behcet"[All Fields] AND "syndrome"[All Fields]) OR "behcet syndrome"[All Fields] OR ("behcet"[All Fields] AND "disease"[All Fields]) OR "behcet disease"[All Fields] | 23 |

Supplementary file 1. Search strategy consisted of ‘Behcet disease’ OR related mesh terms AND ‘Osteonecrosis’ OR related mesh terms.
